# 

**Published:** 1889-04-20

**Authors:** 


SATURDAY, APRIL 20th, 1889.

**"Rocks Ahead"** - - - - - 33

**The Student's Column.**—A complete Compendium of the Science and Art of Surgery - - - - 34

**The Doctor's Pulpit.**—Man and his Enemies - - - 35

**What Must I do to Get Well?** - - - - 36

**Twelve Hundred Miles under Difficulties.** By Daniel Chamier - - - - 37

**Within the Wards.**—Vivisectors and their Work—The Making of New Features - - - 38

**Medicine Men of the World** - - - - 39

**Notes and News** - - - - 40

**Everybody's Page** - - - - 42

**Annotations.**—Weak Hearts—Nervous Headaches—Progress by "Natural Selection" - - - 43

**Life in Modern Asylums** - - - - 44

**"Her Heart's Mistake."** By E. D. Cumming, Author of "An Ordeal by Silence" - - - 45

**Editor's Letter-box.**—Abstainers and Life Insurance—Neglected Men's Wards - - - 47

**Scraps and Gleanings** - - - - 48

**Amusements and Relaxation** - - - - 48

**The Guide to Stretcher and Bearer Company Drill** 48

**EXTRA SUPPLEMENT—THE NURSING MIRROR.**

**En Passant.**—A Hint for Everyone—District Nurses at Bristol—Nursing Lectures at Portsmouth—Maternity and Dispensing Classes—Nurses and Lunatics—Consecrated Women - - - ix
**The Nursing and Management of the Insane.** By T. Duncan Greenlees, M.B. - - - x
**Nursing in the Soudan** - - - - x
**Vaccination and its Opponents** - - - xi
**Everybody's Opinion.**—Leicester Infirmary—Private Institutions—Nursing Institutions - - - xii
**Appointments - Vacancies** - - - xii
**Off-Duty Hours for Nurses** - - - xii

